# *Past the Pandemic*: a virtual intervention supporting the well-being of healthcare workers through the COVID-19 pandemic

**DOI:** 10.3389/fpsyg.2023.1227895

**Published:** 2023-10-26

**Authors:** Amanda M. Millar, Amanda M. Doria, Leslie M. Choi, Laura McGladrey, Korrina A. Duffy, Steven J. Berkowitz

**Affiliations:** ^1^Department of Psychiatry, University of Colorado School of Medicine, Aurora, CO, United States; ^2^College of Nursing, University of Colorado School of Medicine, Aurora, CO, United States

**Keywords:** mental health, burnout, resiliency, online, healthcare workforce, doctors, nurses, hospital staff

## Abstract

To decrease burnout and improve mental health and resiliency among doctors, nurses, and hospital staff during the COVID-19 pandemic, the University of Colorado partnered with ECHO Colorado to offer the state’s healthcare workforce an interactive, psychoeducational, and online intervention that encouraged connection and support. The series utilized the Stress Continuum Model as its underlying conceptual framework. Between July 2020 and February 2022, 495 healthcare workers in Colorado participated in the series across eight cohorts. One-way repeated measures ANOVAs were performed to test for differences in pretest and posttest scores on series’ objectives. Healthcare workers showed significant improvement from pretest to posttest in (1) knowing when and how to obtain mental health resources, *F*(1, 111) = 46.497, *p* < 0.001, (2) recognizing of the importance of being socially connected in managing COVID-related stress, *F*(1, 123) = 111.159, *p* < 0.001, (3) managing worries, *F*(1, 123) = 94.941, *p* < 0.001, (4) feeling prepared to manage stressors related to the pandemic, *F*(1, 111) = 100.275, *p* < 0.001, (5) feeling capable in dealing with challenges that occur daily, *F*(1, 111) = 87.928, *p* < 0.001, and (6) understanding the Stress Continuum Model *F*(1, 123) = 271.049, *p* < 0.001. This virtual series showed efficacy in improving the well-being of healthcare workers during a pandemic and could serve as a model for mental health support for healthcare workers in other emergency response scenarios.

## Introduction

It is well established that mental health symptoms and disorders increased among healthcare workers during the COVID-19 pandemic, with substantial increases documented for anxiety (22% pre-pandemic to 31% during; Giusti et al., 2022), depression (17% pre-pandemic to 36% during; Giusti et al., 2022), insomnia (45% pre-pandemic to 64% during; McCall et al., 2021), and posttraumatic stress symptoms (13% pre-pandemic to 37% during; Giusti et al., 2022). Even for healthcare workers who did not experience a mental health disorder during the pandemic, burnout remained a critical concern. Burnout is defined as a state of emotional, physical, and mental exhaustion caused by excessive and prolonged stress (Moll et al., 2022) and has increased substantially among healthcare workers during the pandemic, with one study showing increased rates from 36% pre-pandemic to 52% during the pandemic (Giusti et al., 2022). Healthcare workers experiencing burnout provide lower quality patient care, increasing the risk of patient death and medical malpractice lawsuits (Dyrbye et al., 2017).

Healthcare workers who are burned out exit the field at alarmingly high rates, which is very costly and burdensome for the healthcare system (Dyrbye et al., 2017; Moran et al., 2020). Nurses in particular experience high rates of burnout due to overwhelming workloads, and feeling underpaid and underappreciated. These circumstances were only exacerbated during the pandemic, when 3.3% of the nursing workforce left the field (Martin et al., 2023). While prior to the pandemic both doctors and nurses already had higher rates of suicidal ideation and suicide than the general population (Davis et al., 2021), during the pandemic the World Health Organization acknowledged an association between exhaustion and suicidal thoughts in healthcare workers, and a 25% increase in anxiety and depression worldwide (COVID-19 Pandemic Triggers, 2022). The consequences of increased mental health problems and burnout due to the pandemic cannot be overstated or overlooked.

At the time of this writing, new variants of COVID-19 continue to spread (The Lancet, 2023), highlighting the critical need for interventions for healthcare workers that are aimed at preventing burnout and enhancing coping strategies (Moran et al., 2020). Web-based interventions are particularly effective during a pandemic as they are highly accessible to healthcare workers and promote peer support and social connection, reducing feelings of isolation without risk of virus transmission (Ye, 2021). A study found that healthcare workers who received a self-paced, web-based intervention during the COVID-19 pandemic felt that the most helpful aspects were the normalization of what they were feeling as well as psychoeducation on self-care strategies and emotional management techniques (Blake et al., 2020).

The Stress Continuum Model is an efficacious model for assessing psychological well-being developed by the US military. In this model, the psychological stress of active-duty personnel is understood as potentially causing injury, and four stages of psychological well-being are defined – “ready” (green zone), “reacting” (yellow zone), “injured” (orange zone), and “ill” (red zone) – with the latter three stages indicating progressively more impaired states of stress (Nash, 2011). Once someone self-reports their current color zone, appropriate interventions can be identified and utilized. Notably, stress injury is considered a physical injury, highlighting the importance of understanding the physical toll stress takes on our bodies while also diminishing our emotional and psychological capacity to cope. Similar to soldiers in conflict, healthcare workers during the COVID-19 pandemic were deployed into high-risk roles and faced uncertainty, illness, and death for an indeterminate length of time, making the Stress Continuum Model especially applicable for healthcare workers within the context of the pandemic (Morganstein and Flynn, 2021). The model uses language that creates awareness of one’s stress by assessing deployment-related stress, or in this case, pandemic-related occupational stress. This process empowers healthcare workers to intentionally respond to their stress levels with the goal of preventing or mitigating stress injury (burnout) and its impact on their personal and professional well-being.

Recognizing the immediate need for a mental health intervention targeting healthcare workers in the initial stages of the pandemic, the University of Colorado School of Medicine’s Department of Psychiatry collaborated with ECHO Colorado to develop an interactive, virtual, didactic series aimed at addressing the stress and anxiety unique to healthcare workers during the pandemic. The intervention incorporated concepts and terminology from the Stress Continuum Model. A team of licensed professional counselors, psychiatrists, psychologists, nurse practitioners, and licensed clinical social workers provided psychoeducation and support free of charge to eight cohorts of Colorado healthcare workers. The series was offered to direct patient care providers (e.g., nurses, doctors, technicians) as well as ancillary workers in the healthcare system, as all those in healthcare were impacted by the pandemic. This is a novel approach given that interventions tend to target nurses and doctors exclusively (Dyrbye et al., 2017). To our knowledge, our program is the only web-based, synchronous, population-based intervention in Colorado offered free of charge to healthcare workers across the evolving phases of the pandemic.

Here, we report on the outcomes of the intervention during the program evaluation period from July 2020 through February 2022. Although the pandemic is ongoing at the time of this writing, we refer to it in the past tense for clarity as well as to represent the evaluation period.

## Methods

Between July 2020 and February 2022, a psychoeducational, interactive, and virtual didactic series called *Past the Pandemic* was offered free of charge to eight cohorts of healthcare workers in Colorado to name and address stress and improve coping. The series was intended to target the challenges healthcare workers faced during the COVID-19 pandemic. Healthcare workers who provided direct patient care (e.g., doctors and nurses) as well as those with ancillary jobs within the healthcare system (e.g., social workers, front desk staff, care coordinators, public health consultants, and environmental services) were eligible to enroll in the series. The program was funded by a Substance Abuse and Mental Health Services Administration (SAMHSA) Emergency Grant awarded to Colorado and was implemented through a partnership between the University of Colorado’s Department of Psychiatry and ECHO Colorado. The University of Colorado’s Department of Psychiatry provided clinical expertise, designed the curriculum, and facilitated sessions whereas ECHO Colorado provided technological assistance, support and communication with participants, and data collection to evaluate the success of the program. The program was marketed through University of Colorado and ECHO email blasts, featured in a local news segment in December 2021, and promoted through programmatic outreach efforts. Participants registered for *Past the Pandemic* via an online link. Sessions took place over Zoom and relied heavily on the chat and poll functions to promote connection between participants by unifying, validating, and normalizing shared struggles and concerns. In addition, participants had the option to attend resource rooms led by a mental health professional, which provided an opportunity to connect, discuss stress using a common language, and implement coping strategies.

For Cohorts 1–4, the series consisted of eight weekly sessions: (1) Stress and the human machine: Impact of stress on mind, body, and living a life you love; (2) Digging deeper: How the biology of stress informs burnout prevention; (3) Staying connected: Communication, relationships, and cultivating strength; (4) Back to the basics: Balancing nutrition, sleep, and movement; (5) Using mindfulness practices to approach burnout, stress, and uncertainty; (6) Using your SMART brain towards parenting and relationship struggles; (7) Managing what we have lost: Mourning, growing, and making meaning; and (8) Caring for yourself and your patients in the midst of uncertainty. Based on feedback from participants, program leadership decided to shorten the series from eight to six sessions to make it more accessible, beginning with Cohort 5 (June–August 2021). Therefore, for Cohorts 5–8, content was either condensed and combined with another session or eliminated altogether, particularly for content that was less relevant as the pandemic-related stressors shifted (e.g., the topic of uncertainty became less important as the pandemic persisted and vaccines became available and physical distancing restrictions lifted).

### Statistical analyses

We categorized healthcare workers into three categories by profession: doctors/providers, nurses/technicians, and ancillary healthcare professionals. The “doctors/providers” category included participants who listed their degree/profession as either Doctor of Medicine (MD), Osteopathic Medicine (DO), Nurse Practitioner (NP), Physician Assistant (PA), Doctor of Philosophy (PhD), Doctor of Psychology (PsyD), Doctor of Medicine in Dentistry (DMD), or Doctor of Dental Surgery (DDS). When more than one degree was listed, the higher degree was used. The “nurses/technicians” category included participants who listed their degree/profession as either Registered Nurse (RN), Licensed Practical Nurse (LPN), Registered Dental Hygienist (RDH), or Medical Assistants (MA). The “ancillary healthcare professionals” category included participants who listed any other healthcare degrees or professions than the ones listed above (e.g., Licensed Clinical Social Workers [LCSW], public health professionals, practice management, administrative staff, and nutritionists). Participants were also included as “ancillary professionals” in cases when no degree or profession was indicated, but the participant listed a healthcare setting as their organizational setting (e.g., a clinic, health and human services agency, school of medicine). Participants were considered missing for professional category analyses if (1) their degree was either not one of the degrees listed above or was left blank, (2) if their profession was not indicated, and (3) if their organizational setting was not indicated. For example, if a participant indicated they had a Master of Arts degree but did not list their profession or organizational setting, they were considered “missing” for their professional category because we could not determine in which healthcare professional category they belonged.

To understand whether the distribution of demographic variables [participant sex (male vs. female), geographic region (urban vs. rural), and direct patient care (provided vs. did not provide)] differed from chance within each professional category, we ran Chi-Square Tests of Independence for each demographic variable crossed with each professional category, which were recoded as dichotomous variables (e.g., doctors/providers: yes vs. no). If model assumptions were met, then Pearson’s Chi-Square was reported. If at least one cell had an expected count of less than five, then model assumptions were violated, and Fisher’s Exact Test was reported instead.

To examine whether the outcomes targeted by the program improved from baseline, we ran a one-way repeated measures ANOVA of pretest and posttest scores for each outcome: (1) knowing when and how to obtain mental health resources, (2) recognizing the importance of being socially connected in managing the COVID-19 crisis, (3) managing worries, (4) feeling prepared to manage stressors related to the COVID-19 pandemic, (5) feeling capable in dealing with challenges that occur daily, and (6) understanding the Stress Continuum Model. This allowed us to test whether posttest (measured after the completion of the program) differed statistically from pretest (measured at baseline).

Consistent with research showing that nurses (Brooks et al., 2018; Lai et al., 2020; Shechter et al., 2020; Croghan et al., 2021), female healthcare workers (Lai et al., 2020; De Kock et al., 2021), and healthcare workers in urban settings (Kelly et al., 2022) experience higher rates of mental health problems and burnout, in our analyses, we considered whether these demographic variables predicted who benefited most from the intervention. To test whether demographic variables [professional category (doctors/providers, nurses/technicians, ancillary), participant sex (male vs. female), geographic region (urban vs. rural), and direct patient care (provided vs. did not provide)] predicted which healthcare workers would benefit more from the program, we ran a mixed model ANOVA for each outcome with the demographic variable as the between subject factor and time (pretest vs. posttest) as the within subject factor. For each demographic variable, we ran a separate model for each outcome.

## Results

The eight cohorts of *Past the Pandemic* had 590 participants who attended at least one session. Although *Past the Pandemic* was marketed to healthcare workers in Colorado, healthcare workers across the country participated in the program. Participants (*n* = 95) from other states were excluded from analyses as the program was developed and intended for in-state healthcare workers (*N* = 495).

*Past the Pandemic* participants were more likely to identify as white (77%) than the general population in Colorado (based on Colorado Census data: 68% white) and were more likely to be female (92%) than the national healthcare workforce (based on national Census data: 76% female). However, because answering the demographic questions was optional, rates of missing data for each demographic question were high (>55%).

**Figure 1:**
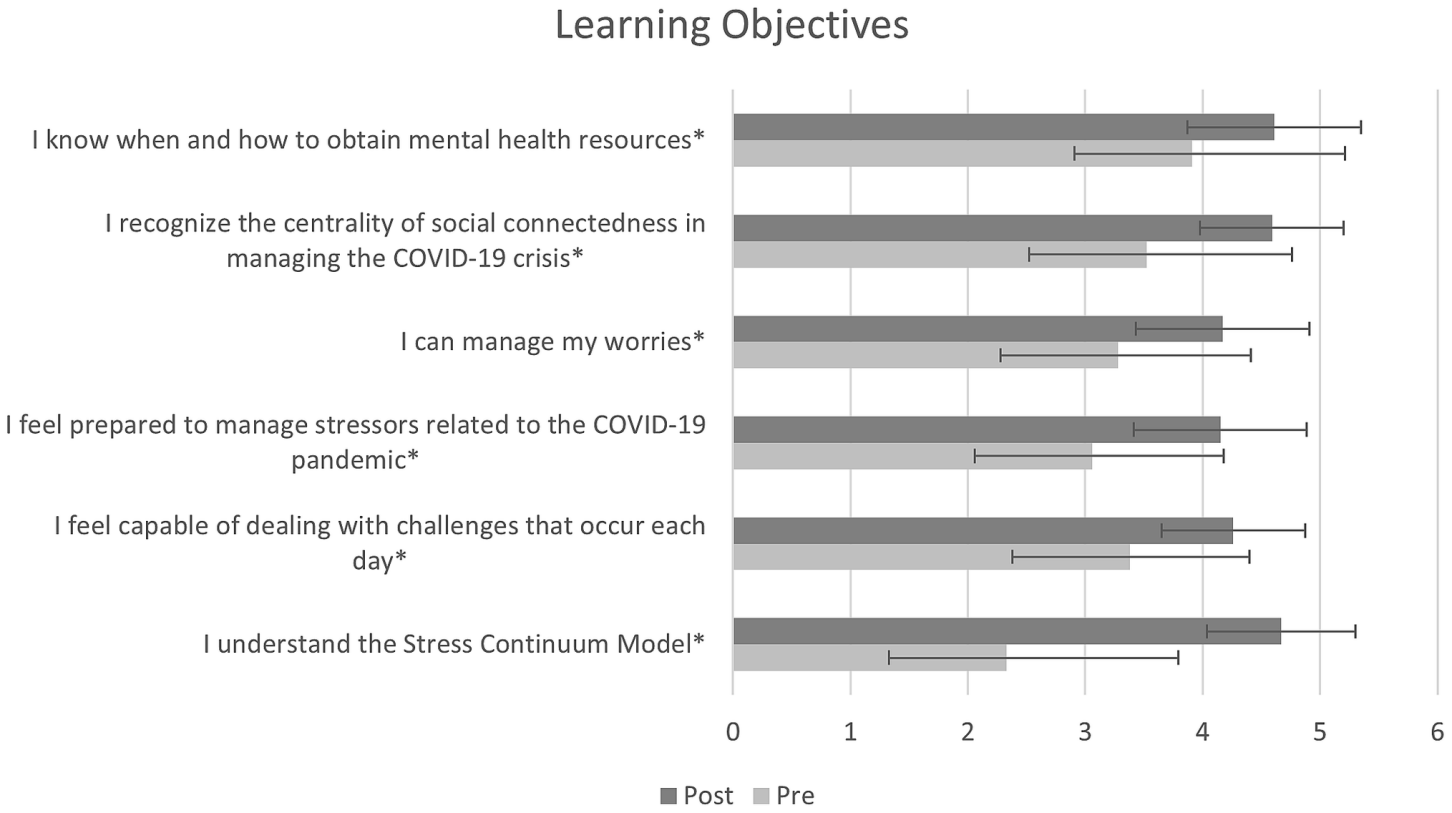
Participants completed questions to measure the impact of the program at baseline and at the end of the series. Participants showed significant improvement in all learning objectives (^*^p < 0.001).

Across all six learning objectives, healthcare workers showed significant improvement from pretest to posttest. Analyses showed significantly increased (1) knowledge of when and how to obtain mental health resources, *F*(1, 111) = 46.497, *p* < 0.001, (2) recognition of the importance of being socially connected in managing the COVID-19 crisis, *F*(1, 123) = 111.159, *p* < 0.001, (3) management of worries, *F*(1, 123) = 94.941, *p* < 0.001, (4) preparedness to manage stressors related to the COVID-19 pandemic, *F*(1, 111) = 100.275, *p* < 0.001, (5) capability in dealing with challenges that occur daily, *F*(1, 111) = 87.928, *p* < 0.001, and (6) understanding of the Stress Continuum Model *F*(1, 123) = 271.049, *p* < 0.001 (see Figure 1). In addition, we tested whether any of our demographic variables (professional category, geographic region, direct patient care, and participant sex) moderated any of the effects reported above to consider whether certain demographic groups benefitted more from the program. However, none of our effects differed significantly by demographic group (*p*s > 0.05).

## Discussion

Our results indicate that after our live, virtual intervention, *Past the Pandemic*, healthcare workers showed significant improvement and confidence in feeling able to manage worries, stressors, and daily challenges during the pandemic, acquire mental health resources, recognize the importance of social connection, and understand the Stress Continuum Model. No demographic group that we assessed (professional category, geographic region, direct patient care, and participant sex) benefited more from the intervention, underscoring its widespread utility and effectiveness among all types of healthcare workers.

During the COVID-19 pandemic, healthcare workers were at increased risk for mental health challenges, partially due to being inadequately prepared to handle the trauma they were exposed to (Moreno et al., 2020; Dohrn et al., 2022). Intervention design that resembles disaster training and leverages technology while emphasizing coping methods, self-care, and support from colleagues can be particularly efficacious for healthcare workers’ well-being (Moreno et al., 2020). The focus of the online *Past the Pandemic* series was to help healthcare workers build skills to mitigate stress and manage the complex emotions related to the pandemic. The intervention’s design allowed participants and facilitators to connect weekly to name, validate, and develop coping strategies to address the struggles healthcare workers faced during the pandemic. By offering consecutive sessions, each cohort had time to build trust, forge a community of support, and instill a sense of reliability and predictability, during a time when those resources felt scarce. This offering provided facilitated opportunities for peers to support each other and normalize their experiences, which was especially valuable as the pandemic (and society’s response to it) continued to evolve – with case and death surges, personal protective equipment shortages, hospital overload, staffing shortages, lockdowns, mask mandates, restrictions lifting, and vaccines becoming available.

**Figure 2:**
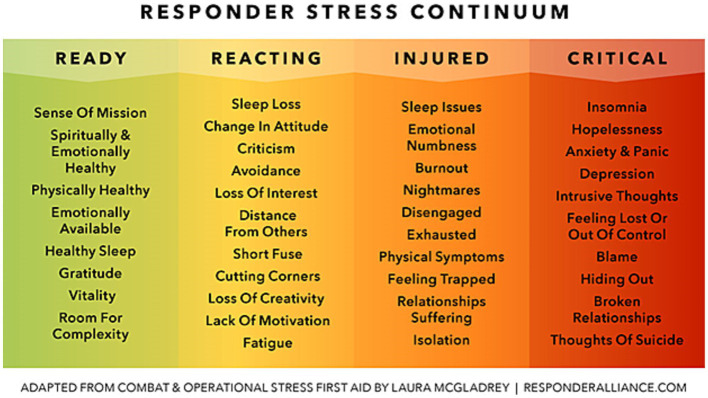
Adapted responder stress continuum framework used for *Past the Pandemic.*

Research has suggested that the Stress Continuum Model could be a beneficial framework to address stress for healthcare workers (Lippy, 2019; Marco et al., 2020; Ganzel et al., 2021; Major et al., 2021; Morganstein and Flynn, 2021), yet to our knowledge, our program was the first to test the efficacy of the Stress Continuum Model within an intervention for healthcare workers. The Responder Alliance^1^ adapted the Stress Continuum Model in order to service search and rescue teams, ski patrol, and National Park Service first responders in Colorado. Their adaptations included using the term “critical” instead of “ill” (red zone), emphasizing the importance of specific behavioral patterns like sleep, and explicitly discussing suicidal ideation (Responder Alliance, 2023). Additionally, their adapted model emphasized self-awareness, self-deployment, and self-assessment, which is a departure from the original model used by leaders to categorize and externally evaluate colleagues subordinate to them. We used this adapted model, the Responder Stress Continuum, as the framework for *Past the Pandemic*, which enabled the healthcare workforce to develop a shared language on the biology of stress and stress injury as an occupational injury (e.g., burnout) as well as learn how to examine and respond to one’s own emotional and physical states (see Figure 2). The Responder Stress Continuum framework allows for early recognition and mitigation of stress, through naming the predictable and modifiable nature of occupational stress exposure. All *Past the Pandemic* sessions asked participants to share their current zone to normalize the often-difficult conversation about one’s personal experience of lived and ongoing stress. The content delivered in *Past the Pandemic* addressed relevant themes for healthcare workers during the pandemic, such as managing stress reactions, experiencing grief, improving resiliency through staying connected, receiving and giving support, getting adequate sleep, and being mindful in everyday life.

*Past the Pandemic* was a unique support program that evolved to respond to themes most relevant at any phase of the pandemic. As we saw new needs of healthcare workers emerge, we modified our program to respond to those needs using an adaptive approach to update the curriculum. Early in the pandemic, for example, we narrowed our initial curriculum from eight to six sessions to focus on the most important content and reduce participant burden. As the pandemic continued, we learned that healthcare workers needed even more flexibility with the timing of when they could access program content, so we provided an option on our website to view video content at their own pace. While we strongly recommended that participants attend the first two *Past the Pandemic* sessions live, they were not required to attend all the sessions thereafter and could view recordings of the sessions as they desired. After the program’s first year, the website^2^ was created to host session videos, resources, and a toolkit. These continual modifications and additions allowed participants to attend sessions or view resources when they had the capacity to do so, without imposing a sense of pressure.

Since feelings of self-efficacy and social connectedness have been shown to mediate the stressful impacts of trauma exposure for healthcare workers (Shoji et al., 2014; Morganstein and Flynn, 2021), the *Past the Pandemic* program was designed to provide healthcare workers with strategies to improve self-efficacy and social connectedness. For later cohorts, we shared with participants the *Past the Pandemic* Toolkit, a 42-page compilation of exercises, worksheets, and resources. The toolkit allowed participants to apply the curriculum to their specific struggles and concerns, implement strategies they learned (e.g., enhancing self-efficacy) as well as share them with coworkers and family (e.g., enhancing social connection). While we have not yet formally evaluated the toolkit, it is one of the most visited pages on our website, and in qualitative evaluations, participants have endorsed it as a helpful and timely resource.

### Limitations

Since we thought it was important to support not only healthcare providers in hospital settings but all professionals who work in healthcare settings (including administrators, care coordinators, social workers, and outreach coordinators), our cohorts included an array of professionals who were not healthcare providers (categorized as “ancillary”) in line with recommendations to reach all who work in healthcare settings whose lives and workplaces have been affected by the pandemic (Morganstein and Flynn, 2021). However, future interventions could more intentionally target those professions that are typically not targeted for such interventions, like those working in food or custodial services. In addition, future interventions could also be tailored to professionals in rural healthcare settings, who may have different challenges related to economic disadvantage, geographical isolation, supply chain issues, and provider shortages (O'Sullivan et al., 2020).

Our current program evaluation is limited to assessing the program’s impact immediately after completion. However, in a future program evaluation, it would be beneficial to follow-up with participants longitudinally to assess lasting knowledge acquisition, behavioral change, job retention, and overall well-being. In addition, the current program evaluation relied exclusively on participant self-report, which could result in response bias due to demand characteristics of such questions (i.e., in which the context makes participants aware of the way they are expected to respond). Furthermore, we did not measure burnout, depression, anxiety, and PTSD pre and post intervention although it would be helpful to know whether our intervention impacted such outcomes. Future pandemic response programs would likely benefit from collecting more thorough participant data. Because we designed our program early in the pandemic, our primary goal was to respond to the increasingly troublesome and frequent burnout symptoms apparent in our healthcare colleagues (e.g., stress, exhaustion, irritability, negativity, loss of motivation); thus, having a robust dataset was of secondary concern. In addition, we did not anticipate the longevity of the program, which we continued to run when pandemic stress became chronic.

During the COVID-19 pandemic, healthcare workers have faced unprecedented risk and incidence of burnout and mental health disorders, particularly anxiety, depression, insomnia, posttraumatic stress, and even suicidal ideation. Healthcare workers who become aware of their stress and its impact and use coping strategies to decrease their burnout and improve their resiliency in the face of an ongoing pandemic have the potential to find fulfillment and purpose in their jobs, remain in their healthcare positions, save the healthcare system money, and, most importantly, provide better patient care. *Past the Pandemic* was our solution to the potential fallout of the pandemic for healthcare workers and showed initial efficacy in helping healthcare workers cope. The skills, strategies, and resiliency learned through our program may not only help healthcare workers during a disaster response but are also applicable beyond the pandemic and can be incorporated into everyday life.

## Data availability statement

The raw data supporting the conclusions of this article will be made available by the authors, without undue reservation.

## Ethics statement

Ethical review and approval was not required for the study on human participants in accordance with the local legislation and institutional requirements. Written informed consent from the patients/participants or patients/participants legal guardian/next of kin was not required to participate in this study in accordance with the national legislation and the institutional requirements.

## Author contributions

AM, KD, and SB contributed to the conception and design of the study. AM organized the database and wrote the first draft of the manuscript. AM and KD performed the statistical analysis. AD, LC, and LM wrote sections of the manuscript. All authors contributed to the article and approved the submitted version.

## Footnotes

^1^ www.responderalliance.com

^2^ https://pastthepandemic.org
